# Optimal Deployment of FiWi Networks Using Heuristic Method for Integration Microgrids with Smart Metering

**DOI:** 10.3390/s18082724

**Published:** 2018-08-19

**Authors:** Esteban Inga, Miguel Campaña, Roberto Hincapié, Oswaldo Moscoso-Zea

**Affiliations:** 1 Electrical Engineering, Universidad Politécnica Salesiana, Quito EC170146, Ecuador; mcampana@ups.edu.ec; 2 Department of Telecommunications, Universidad Pontificia Bolivariana, Medellín 050031, Colombia; roberto.hincapie@upb.edu.co; 3 Faculty of Engineering, Universidad Tecnológica Equinoccial, Quito EC170147, Ecuador; omoscoso@ute.edu.ec

**Keywords:** optimization, smart metering, IoT, microgrid, heuristic, sensor networks

## Abstract

The unpredictable increase in electrical demand affects the quality of the energy throughout the network. A solution to the problem is the increase of distributed generation units, which burn fossil fuels. While this is an immediate solution to the problem, the ecosystem is affected by the emission of CO_2_. A promising solution is the integration of Distributed Renewable Energy Sources (DRES) with the conventional electrical system, thus introducing the concept of Smart Microgrids (SMG). These SMGs require a safe, reliable and technically planned two-way communication system. This paper presents a heuristic based on planning capable of providing a bidirectional communication that is near optimal. The model follows the structure of a hybrid Fiber-Wireless (FiWi) network with the purpose of obtaining information of electrical parameters that help us to manage the use of energy by integrating conventional electrical system with SMG. The optimization model is based on clustering techniques, through the construction of balanced conglomerates. The method is used for the development of the clusters along with the Nearest-Neighbor Spanning Tree algorithm (N-NST). Additionally, the Optimal Delay Balancing (ODB) model will be used to minimize the end to end delay of each grouping. In addition, the heuristic observes real design parameters such as: capacity and coverage. Using the Dijkstra algorithm, the routes are built following the shortest path. Therefore, this paper presents a heuristic able to plan the deployment of Smart Meters (SMs) through a tree-like hierarchical topology for the integration of SMG at the lowest cost.

## 1. Introduction

Nowadays, the need to integrate modern technologies in conventional electrical distribution systems is of crucial importance in terms of optimization, security, confidence, reliability and energy efficiency [1]. One of the critical issues in power distribution systems is the uncontrollable increase in demand. This is mainly due to the increase in consumers and the increasingly high dependence on electricity for heating and cooling. Therefore, these factors are enablers of significant fluctuations in the rate of consumption of electrical energy. With the increase in demand at peak hours, there is a need for more generation plants to avoid voltage drops and the decrease in the quality of the electrical energy. As a result, institutions should encourage Demand Side Management (DSM), which becomes viable by implementing robust bidirectional communication systems [2]. These systems need appropriate hybrid topologies to allow the communication network to provide the user with reliability and safety on the use of information [3]. This approach opens a path toward the existence of an Intelligent Electric Network (IEN). An IEN is possible thanks to the use of communications to obtain data on the intrinsic components of a network (data obtained from producers to consumers). This contributes to our economic and environmental health [4]. The information obtained from the network will be collected by Smart Meters (SMs) [5] spread over the area of interest, and their locations will be fixed [6]. The conventional electricity meters must necessarily be replaced by SMs, since they will be able to communicate with diverse types of electronic devices [7] distributed in the conventional network. Each SM will not only be able to receive and transmit information of electrical parameters as active and reactive power, but will also have the ability to run events, such as reconnection, disconnection, sensing the theft of electricity supply, integration of Distributed Renewable Energy Sources (DRES) and the proper management of energy resources in each individual household. The measurements can be collected without the need to visit the facilities of the customer. This may be carried out in intervals of time of 15, 30 or 60 min. The analysis of these data supports analysts to improve the operation, planning, control and supervision of the conventional electric network [8,9]. Additionally, this analysis gives us sufficient data to contribute to forecasting home loads and the grouping of each load profile [10].

### 1.1. Importance of the Two-Way Communication System in Smart Grids

It is believed that DRES play a significant role in the reduction of greenhouse gas emissions [11,12,13,14]. This improves the availability of the energy resource, increasing the efficiency and the quality of the supplied energy [15]. DRES is essential for the sustainability of the conventional electrical system and is part of the solution to the uncertainty of the demanded load. DRES are not easy to use, as they increase the complexity of the system [16,17].

The implementation of bidirectional communication technologies (low-cost and low-consumption) leads us to integrate the concept of the Smart Grid (SG) described in [18,19,20]. In an electrical network, an SG is conceived of as a network that can deliver electricity in a controlled manner, from the points of generation to the active consumers [21]. In addition, SG will adjust the amount of energy generated according to the real-time demand of consumers, thus avoiding the excess of generation and covering most of the required demand [22]. Therefore, changes in supply and demand require a more intelligent system that can handle the increasingly complex electrical network [15].

As a result, an efficient design of SGs tackles three elements: communication, control and optimization [16,19,23,24]. In this paper, we will give special attention to smart metering of electrical energy with the purpose of obtaining accurate information from electricity consumption and in this way run energy management processes at the lowest cost; enabling us to not only automate the distribution in energy, but in addition, allowing us to introduce the use of DRES to SG, granting enforcement and control of the system. The observance of the electrical system will allow us to know the instantaneous supply and demand. This is with the aim of predicting energy consumption [23].

Advanced Metering Infrastructure (AMI) allows a two-way communication in which SMs must be able to send the information collected in the analysis tools and receive operating commands from the central office [25,26,27]. In order to avoid communication conflicts, it is very important to establish communication standards that allow the interoperability between different electronic equipment, as suggested in [12,16].

This paper proposes the implementation of a heuristic that provides a near optimal route map of SMs in a georeferenced area. The heuristic will be able to form clusters [28,29,30,31], optimize resources and depict a route map observing parameters of capacity and coverage. In [23,29,32], some examples of the advantages of the groupings are described. These groupings are used for the following reasons: to optimize the use of bandwidth, to optimize the use of energy, to reduce overhead costs, to increase connectivity, to stabilize the network topology, to decrease delays and load balancing. This will reduce the Free Space Path Loss (FSPL) and decrease the end to end delay, ensuring real-time communications for optimal operation of the FiWi network in smart cities.

### 1.2. FiWi Network Architecture

A hierarchical clustering method using a topology-type tree will be used in this paper. This method is a fundamental operation in the deployment of SMs [33,34]. The paper from [35] stated that an optimal conformation of the clusters is determinant in order to minimize the end to end delays of each cluster. The knowledge of the end to end delays in a wireless network can be used to deduce the available bandwidth. There are some techniques based on the observation of the delays to detect the point at which the delays begin to increase and, in this way, to determine the bandwidth. The results show that it is feasible to obtain reliable estimates under certain circumstances, such as: different packet sizes, wireless link speeds and channel noise [36,37,38]. Therefore, in the present paper, the bandwidth is considered through the delays, not as a restriction of the problem, but as a parameter that can be calculated to size the equipment and achieve a robust wireless communication network that guarantees the transmission of the information in a safe and reliable way. Finally, the required bandwidth depends on: the packets transmitted from a source node to a destination node, the traffic they generate, the frequency at which they are transmitted, the point-to-point distance and the number of jumps. Therefore, the maximum data transfer range of a physical communication link is proportional to its bandwidth [39,40].

There are two types of hierarchical groupings: binder and divisive [41,42]. The binder method starts by placing each object in its respective cluster and then merging the groups into larger clusters, until all the objects are in a unique cluster or certain conditions are met. The divisive hierarchical grouping method is not limited to grouping into a balanced cluster or into a cluster of the same length, as some conventional methods do. Some of these conventional clustering methods are k-means, k-medoids and mean-shift [43].

**Figure 1:**
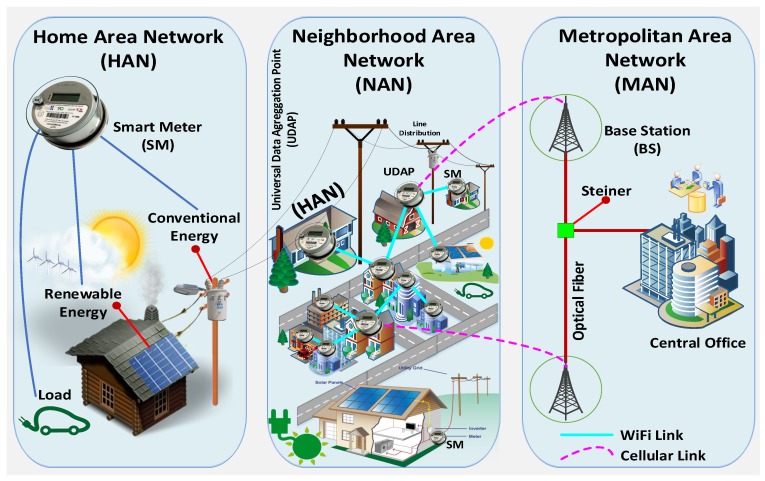
FiWi network architecture for the efficient integration of smart meters. Source: the authors.

In the present paper, we will form clusters using the binder method. This is a method that allows us to balance the length of the clusters and minimize certain parameters of a communications network such as: end to end delay, FSPL and the ability to link. In the clustering techniques, the SMs are organized into groups. The regular SMs are called cluster members, and a head is selected from the group tagged as the Universal Data Aggregation Point (UDAP). There are three types of generated traffic: intra-cluster, inter-cluster and the existing traffic generated by base stations (BS) toward the central office [29,44,45,46]. These are illustrated in Figure 1 through the use of optical fiber. The members of a cluster cannot send data directly to the BSs, since the UDAP receives data from the SMs members of the cluster, eliminates redundant data and merges the data with the objective of transmitting to the respective BSs.

This paper considers a FiWi network [47] in two stages: The first stage describes a wireless hybrid network [48,49] that articulates cellular technology and WiFi to transport the information from the SMs to the BSs passing by a node of transition UDAP capable of supporting both technologies. This will ensure efficient and effective two-way communications within standardized parameters for the communication [47,50,51,52]. In the second stage, data are merged in the BSs using optical fiber. Following that, a backhaul will be added to transmit the information to the central offices or information management centers. The properties of the UDAP allow receiving data from the SM members of the conglomerate. Following that, it merges the data and transmits the added information to the BSs. A UDAP is an SM with double availability for cellular and WiFi wireless access.

In summary, we make the following contributions in this paper: (1) the proposed heuristic focuses on minimizing the data aggregation cost. This heuristic uses a hierarchical topology capable of reducing transmission delays, contributing directly to minimizing link capacity [53]. This results in significant cost reduction for the implementations at the physical and at the link level; (2) The mathematical optimization model considers the deployment of the FiWi network under planning and scalability over time and space; (3) The model provides a near optimal route map with georeferenced coordinates. This model uses the haversine equation for the calculation of distances. Moreover, the model is able to provide accurate data about the topology of the network and the roadmap for the hybrid near optimal communication path for the deployment in AMI; (4) The heuristic provides answers to the challenges of UDAPs’ placement, identification of target groups, routing, capacity, coverage and reduction of the end to end delay. Hereinafter, the paper is structured as follows.

Section 2 describes the need to update the concept of a “conventional electric network” with the purpose of migrating to the concept of a smart grid, and discusses the importance of AMI for optimal deployment of microgrids. Section 3 sets out the approach to the problem. Section 4 presents the results and simulations. Finally, in Section 5, conclusions are drawn.

## 2. Conventional Network and the Need for Smart Grids

Research in the modeling of residential demand is typically focused on the monthly or yearly data average demand. Little emphasis is put on energy consumptions in a home or appliance in particular in this line of research [54]. Residential consumption represents an important share of the total electricity demand, due to the exponential growth experienced throughout the world. In this context, the prediction of the energy demand of the housing industry is important, as suggested in [15,23]. Consequently, a new concept is introduced: “the demand of the firm”, which refers to the ability to control the individual loads. Moreover, the demand of the firm refers to load management, which means being able to have real-time and smart control of the load. In the conventional electrical system, there are two types of controls, which are: cost control and direct control [23]. Cost control seeks to change the form of the load curve [55] without considering that the consumption of energy increases. This mechanism entails increasing energy prices in peak periods and the application of new rates. The direct control refers to the classic methods of load control involving the increase in energy production when the demand increases [4].

The electricity is generated and distributed on a hierarchical network that has three different subsystems: generation, transmission and distribution. The aggregation of data on each of the subsystems of an electric network is crucial in SG for the control, protection, automatic functioning of interrelated components and the integration of DRES in IEN [56]. DRES are capable of functioning independently or in conjunction with the main electrical network under the concept of microgrids [57,58].

The rapid advances in automation and control generate potential benefits, such as: reducing the consumption of resources, improvements in infrastructure capacity and the coordination of the demand peaks [8,59]. This is mainly due to the introduction of the Information Communication and Technology (ICTs) [60], which has allowed the transformation of the conventional electrical network into an electrical network that ensures the productive interaction among suppliers of power, consumers and other stakeholders, as suggested in [12,15,61,62,63]. Therefore, changes in generation, transmission and distribution systems are inevitable [16].

A smart electrical network should be able to motivate consumers to participate actively in the operations of the network and, as suggested in [23,47,64,65], must be able to withstand attacks to provide a higher quality of power. For the existence of IEN, a large-scale implementation of sensors and measuring instruments is necessary, which have to be able to communicate with each other in order to add data from the state of the network [66]. The services of data aggregation can be structured as a tree, and their goal is to merge data from various sources [22,67]. Finally, the European Commission defines a smart electrical network as: “An electrical network that can integrate efficiently the behavior and actions of all the users in a framework based on rules and priorities for achieving interoperability of devices in a system of smart electrical networks” [63].

### AMI in Microgrids

Nowadays, there are new devices that are capable of processing information in the electrical sector and that access the Internet or adjust the energy consumption based on cost or availability depending on the preferences of consumers. All of this is part of what is called the Internet of Things (IoT). The “things” in SG include sensors [3,68,69], smart devices and the SMs [1,27,68,70]. The devices need to be interconnected in a hierarchical network with adequate levels of quality and reliability. The introduction of SG contributes to providing digital intelligence to the power system network [56]. The benefits associated with these new concepts are: adequate management of the energy resources, reduction of the interruption rates, reduction of the pollution rates in the ecosystem, reduction in the number of interruptions due to problems in the quality of power and lower costs of operations and maintenance [1]. Consequently, one of the main benefits of SG is the intelligent and efficient design of hybrid communication networks, which take into account the congestion of the network, real-time transmission as suggested in [2,47,71] and the concern of reducing the emissions of greenhouse gases [69].

The fast growth of data requires researchers to pay attention to how to handle these data [72]. Therefore, three definitions have to be analyzed: volume, velocity and variety. Volume refers to the large amount of data to be processed; the speed refers to the latency of data transmission; and the variety refers to the different types of data that must be processed [59]. The consumers of energy resources are equipped with SMs that collect the data in real time. AMI receives all data and sends them to Meter Data Management Systems (MDMS) that controls the storage. MDMS is in charge of the analysis of data and provides the information in a useful way [73,74]; in addition, the efficient management of wireless resources is essential to increase the life of the network [75]. AMI is not a technology, but rather a configured infrastructure that integrates a series of technologies to achieve their goals. AMI includes SMs, communication networks, MDMS and the tools to integrate the collected data of software application platforms and interfaces [16,76]. Among the communication technologies used in this paper for extracting and transporting the information are WiFi, cellular and optical fiber.

Optical fiber has dominated by being able to maintain communications over long distances, such as for metropolitan networks (see Figure 1). Additionally, it provides increased bandwidth, low transmission losses and greater tolerance to other cable access technology interference [77]. One of the disadvantages is that it requires a huge cost for a deep penetration of fiber. Therefore, the wireless access networks are a promising technology, since they provide the flexibility of low cost, increase the coverage and robustness and are easy to implement. A disadvantage is that the bandwidth capacity is limited severely [78]. Therefore, considering the advantages of each technology, it was proposed to build a hybrid network technology that includes wireless technology and optical fiber.

The integration of renewable energy resources with small sources of storage leads to the concept of microgrids [74,79]. The uncontrolled integration of microgrids affects the quality of the power, among which, the more important events are sag voltage signals induced by failure defects [80]. Therefore, with the insertion of DRES, the quality of voltage cannot be guaranteed when there is not a communications system to provide timely information of the state of the conventional network. To ensure the quality of voltage in the network, through the integration of microgrids, the voltage levels of the conventional network and the DRES must be resynchronized [81]. This resynchronization can be done by obtaining real-time information of the state of the network. Hence, the key is the integration of an adequate communications infrastructure that allows the aggregation of data and AMI to monitor and control the conventional electrical network. This allows the levels of voltage to be always known when introducing microgrids to run adequate processes of quality energy management.

Therefore, in this paper, we propose a heuristic method capable of providing a roadmap for deployment of an advanced metering infrastructure. This method can be a solution to the sizing problem. In this way, it allows the planning of FiWi communication networks considering certain restrictions. The speed of transmission of data does not intervene in the model as a restriction, but it can be estimated knowing the packet rate, transmission rates and data length generated by the SMs. In this research, these values are taken from the literature. Another calculated parameter that depends directly on the distance is the FSPL. These parameters are referents to determine the importance of the topology and how it affects the network for the minimization of the end to end delay and the losses in the free space. The model minimizes the number of SMs that use cellular technology through the incorporation of WiFi technology. In summary, in this work, we intended to deploy a WiFi communication network optimizing resources through clustering techniques. These techniques are based on a variant of the Prim algorithm and the minimum expansion tree algorithm (Dijkstra). With these algorithms, the adjacency matrix (G) is constructed. This matrix is formed with the existing relations between the different elements of the communication network (SMs, UDAPs, BSs and central office). These elements will form the resulting route map for the optimal integration of DRES. The model includes the connectivity of BS and the central office using a fiber optic link. In this way, the communication resources are integrated into a FiWi network.

Table 1 presents the model and parameters of simulation to be used in this paper.

## 3. Problem Formulation

There are *n* SMs *X* for electrical energy measurements distributed in a georeferenced area *A*, A(n). With Algorithm 1, Nearest-Neighbor Spanning Tree (N-NST), the clusters are formed and using Algorithm 2, Optimal Delay Balancing (ODB), the SM is selected that will become the head of the group (UDAP) *Z*. Each cluster has a capacity to group until *m* SMs. We assume that the maximum range of bidirectional transmission of intra-cluster data is $r_{ds}$ and the maximum range of bidirectional data transmission of inter-cluster data is $r_{db}$. That is to say, any intra-cluster and inter-cluster length, the haversine distance $r_{ni}$ and $r_{ns}$ of which is within $r_{ds}$ and $r_{db}$, respectively, can communicate between each other. The *X* and *Z* that do not reach the maximum haversine distance allowed in a single jump will do so with multiple jumps until being able to transmit the respective data packages. The multiple breaks are restricted by *w*, which is the maximum number of jumps allowed. It is worth mentioning that an SM will not be able to transmit its data directly to the BSs. Therefore, the use of a node of transition UDAP (head of each group) is of vital importance to comply with that function. Since UDAP has physically two slots to hold dual wireless and cellular cards, it is able to receive the information transmitted from the access of single SMs to WiFi technology and merge the information to retransmit these data further to the nearest cellular access BSs. Therefore, the allowed breaks will be performed only between intra-cluster SMs or between UDAPs; mainly to transmit the data to the closest BSs to finally send it, via optical fiber, to the central office where the information will be processed. Consequently, the link between each of the vertices (SMs, UDAPs, BSs, central office) can be represented by an adjacency matrix. This matrix indicates the pairs of vertices that are related or not by a link or edge in the graph. In addition, the adjacency matrix is a binary matrix (0, 1) with zeros in its diagonal. It stores one when there is an edge from the vertex *i* to the vertex *j* and zero when there is no edge.

Initially, all *X* are candidate *Z* with a cost $C_{1}$. Once having identified the clusters and the transition nodes *Z*, the links are created at $C_{2}$ cost. Due to this, it eliminates the need for all X to be Z. This happens because cellular links are deleted at a cost $C_{1}$ and links to WiFi are added at a cost $C_{2}$, ensuring the 100% observability of the SMs deployed. Subsequently, the UDAP merges the data and sends them to the BSs. Once the data are merged in the BSs, they will be transmitted through optical fiber to the central office with a cost $C_{3}$ (see Figure 1). The $C_{1}$, $C_{2}$ and $C_{3}$ variables are identified as unit costs for each type of technology: cellular, WiFi and optical fiber, respectively. In addition, it should be noted that $C_{3}>>C_{1}>>C_{2}$. Table 2 presents a summary of the variables used in the model.

In Equations (1)–(3), the total costs of each technology are expressed: WiFi, cellular and optical fiber:

(1) $$C_{wf}=C_{2}*\sum_{j=1}^{k}\left(s_{j}-1\right)$$

(2) $$C_{cell}=C_{1}*k$$

(3) $$C_{fop}=C_{3}*d_{fop}$$

where $s_{j}$ represents the length of each cluster, *k* is the maximum number of clusters to be deployed in the network and $d_{fop}$ is the required distance of optical fiber to be used in the FiWi network.

In this way, the optimization problem can be expressed as follows:

(4) $$min\;C_{wf}+C_{cell}+C_{fop}$$

subject to:

(5) $$C_{i}\in {ℜ}^{+},\forall \;i=1,2,3.$$

(6) $$\sum_{s,k\;\in \;n}\left(s-1\right)+k=n,\;\forall \;s,k\in n;\forall \;n\in A\left(n\right)$$

(7) $$\sum_{SM\in A(n)}SM=Z_{i,j},\;\forall \;Z\in A\left(n\right)$$

(8) $$\sum_{SM\in A(n)}SM=X_{i,j},\;\forall \;X\in A\left(n\right)$$

(9) $$\sum_{s\in S}S\leq \;m,\;\forall \;S\in A\left(n\right);\forall \;m>1$$

(10) $$X=\sum_{r_{ni}\in r_{ds}}r_{ni}\leq r_{ds},\;\forall \;X\in A\left(n\right)$$

(11) $$Z=\sum_{r_{ns}\in r_{db}}r_{ns}\leq r_{db},\;\forall \;Z\in A\left(n\right)$$

(12) $$d_{fop}\in {ℜ}^{+},\forall \;d_{fop}\neq 0.$$

Equation (4) corresponds to the objective function, which consists of minimizing the costs of implementation on a FiWi network. Equation (5) necessarily asserts that there are three types of costs. Equation (6) presents a restriction of verification, in which it must be satisfied that the sum of WiFi links and the sum of cellular links does not exceed the total number of SMs deployed at A. This ensures that there are no loops within the wireless network.

Equations (7) and (8) enable any SM belonging to A to be able to be a UDAP. The restriction of capacity, of Equation (9), limits the number of intra-cluster SMs that will be able to bring together each cluster. In Equation (10), the maximum radio coverage allowed is restricted to give way to the existence of an intra-cluster link. It is very important to mention that the referential distances that are restricted are those from point to point, which are given between an SM and its respective UDAP; in such a way that all the nodes that comply with the restriction would be able to form part of a cluster by a single jump or multiple jumps. Finally, the model verifies the capacity of the conglomerate and the maximum radio coverage. Therefore, if a node needs more than one jump to transmit the information to the UDAP and if the referential distance allows it, the resulting length would be the sum from the initial node, passing through each transition node, until reaching the UDAP. In Equation (11), the maximum radio coverage allowed is restricted to make way for the existence of inter-cluster links. If the cluster heads (UDAPs) do not connect in a single jump to the base station (due to the coverage radius restriction), they could do it by multiple jumps supported in the transition UDAPs. In such a way, the point-to-point distances that are part of the accumulated distances from the initial node to the destination node will be determined by the maximum distance allowed between the UDAPs and the base station. Finally, Equation (12) expresses that the necessary optical fiber distance must exist, guaranteeing the connectivity between the BSs, toward the central office.

**Algorithm 1** Nearest-neighbor spanning tree: receive (m, $r_{ds}$, w, $\gamma_{i,j}$, $dist_{i,j}$).
1:
$aux_{i,j}\leftarrow dist_{i,j};$
2:
$pairs\left(i\right)\leftarrow \gamma_{i,j};$
3:
s←1;h←0;
4:
**if** $length\left(pairs\right)\neq empty\;\&\&\;r_{ni}\leq r_{ds}$ **then**
5: **while** s≤m∥h≤w **do**6:  flag←1;7:  path←pairs(i);8:  **while** flag==1 **do**9:   add← find next adjacent node index $\left(aux_{i,j}\right)$;10:   path←[pathadd];11:   s=s+1;12:   **if** m≤s **then**13:    flag→0;14:   group←path;15:
dist(group)←inf;
16:
Send(group);



**Algorithm 2** Optimal delay balancing: receive (group, $x_{s}$, $y_{s}$).
1:
**for** i→1:length(group) **do**
2: $x_{UDAP}\leftarrow x_{s}\left[group\left(i\right)\right];$3: $y_{UDAP}\leftarrow y_{s}\left[group\left(i\right)\right];$4: $k^{\prime },\;j^{\prime }\leftarrow Dijkstra\left(group,\left[x_{UDAP},y_{UDAP}\right]\right);$5: $path\leftarrow [k^{\prime },\;j^{\prime }];$6: $G^{\prime }\left(\mathrm{path}\right)=1;$7: α←End to end delay,FSPL;8: Sol←[Solα];9: α←min(Sol);10: index(i)←find(α);11:
UDAP←group(index);
12:
Send(UDAP,α);



Algorithm 3 requires the input of the coordinates $\left(SMs,BS\;\mathrm{and}\;C_{off}\right)$. The coordinates are georeferenced. Therefore, it allows rehearsing a real scenario. Following that, a distance matrix is obtained using the haversine formula between the displayed SMs. Once the distance matrix is identified, a γ vector is created with the pairs of adjacent SMs and is ordered according to the distance between pairs from least to greatest. It is important to mention that the starting criterion for the exploration and construction of the clusters begins from the pair of SMs with the minimum distance. In addition, Algorithms 1 and 2 are iteratively called from Algorithm 3 to obtain the results. Once obtained γ, Algorithm 3 calls Algorithm 1 (N-NST) to solve the wireless network deployment by minimizing the number of UDAPs through a heuristic based on the Prim algorithm. Thus, it is possible to guarantee the coverage of SMs as long as they comply with the restrictions. Recall that one of the objectives is to reduce the use of cellular links (higher cost) and exchange them with WiFi links (lower cost). Firstly, from the γ vector, the SM that has the shortest distance to the BS is selected. This SM is a candidate that could be a UDAP. What has been done brings about a pre-clustering of a wireless network that achieves the connection of SMs forming a tree of minimum expansion. This problem is NP-complete. The end to end delay and the losses in the obtained topology are verified by propagation of the wave in the free space. This topology is recorded in the adjacency binary matrix (G) Subsequently, Algorithm 3 verifies by means of Algorithm 2 whether it is possible to decrease the end to end delay and the losses by propagation of the wave in the free space by means of the intra-cluster modification previously obtained. If this decrease is possible, the algorithm takes the latter as the best solution, otherwise it takes the first option. Therefore, the model iterates and corrects what was originally obtained as a result with Algorithm 1. The model iterates until the objective function (subject to restrictions) is the minimum. It also verifies that there is no option to further reduce the cost of cellular links, delays and losses by propagation of the wave in free space. Finally, once the near the optimum route map with the heterogeneous wireless network (WiFi-cellular) is obtained, the algorithm proceeds to find the minimum route from the BSs to the central office with a fiber optic link. In this way, the route map of a heterogeneous FiWi network is achieved as a final result.

**Algorithm 3** Generate topology: receive ($SM_{\;x,y}$ , $BS_{\;x,y}$, $C_{off\;x,y}$, $r_{db}$, $r_{ns}$, n).
1:
$x_{s}\leftarrow \left[SM_{\;x};\;BS_{\;x};\;C_{off\;x}\right]$
2:
$y_{s}\leftarrow \left[SM_{\;y};\;BS_{\;y};\;C_{off\;y}\right]$
3:
$dist_{i,j}\leftarrow \mathrm{haversine}\left(x_{s},y_{s}\right);$
4:
$\gamma_{i,j}\leftarrow \mathrm{sort}\left(\mathrm{find}\;\mathrm{pairs}\;\mathrm{of}\;\mathrm{nodes}\left(dist_{i,j}\right)\right);$
5:
Algorithm1;
6:
group←return;
7:
$\mathrm{Preliminary}\;UDAP\leftarrow Find(min\left(haversine\left(group,\;BS{\;}_{x,y}\right)\right));$
8:
used←length(group);
9:
temp←group;
10:
**while** used≤n **do**
11: **if** index≠1 **then**12:  índex(temp)=1;13:  used=sum(index);14: $k^{\prime },\;j^{\prime }\leftarrow$ Dijkstra(group,UDAP);15: $G(tmp\left(k^{\prime }\right),\;tmp\left(j^{\prime }\right))=1;$16:
β←Delay end to end,FSPL;
17:
Send←group;
18:
Algorithm2;
19:
(UDAP,α)←return;
20:
sol←Siftoutthebestsolutionbetweenvectorsβandα;
21:
path←find(sol)
22:
G(path)=1;
23:
**if** $r_{ns}\geq r_{db}$ **then**
24: G(path)=0;25:
LinkUDAPtothenearestBS;
26:
FindminimumpathbetweenBSstothecentraloffice;



## 4. Results

**Figure 2:**
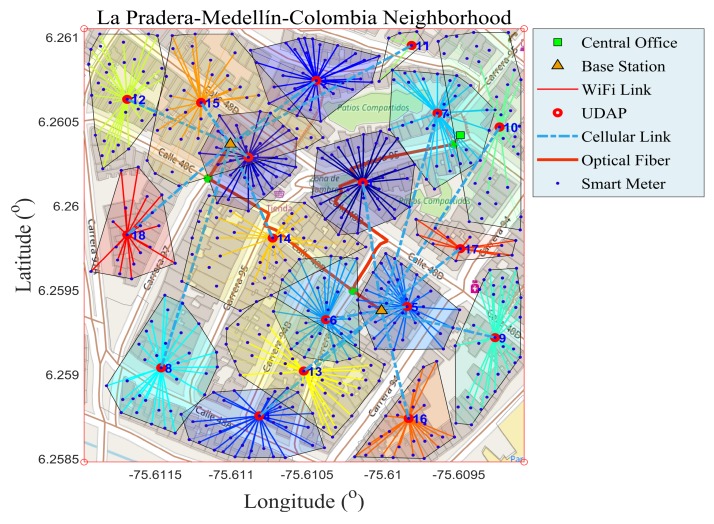
Near optimal deployment of SMs using Fiber-Wireless (FiWi) network. Source: the authors.

The near optimal route map on an advanced measurement infrastructure under the concept of FiWi network allows analysts to know the state of the conventional electrical network for the optimal integration of microgrids and is presented in Figure 2. By having a georeferenced route map, we have all the information required to run the actual deployment, and more importantly, we can account for each of the resources required for planning, implementation, economic assessing and FiWi network operability. In Figure 2 is depicted the existence of a multi-jump intra-cluster, for securing 100% coverage of each of the SMs in the area of interest. It is very important to point out that each cluster of the present paper is formed with a method that is different from the conventional clustering methods (k-means, k-medoid and mean shift). The method that was developed to achieve the goals of the research proposes the application of Algorithm 1, N-NST. Since it is capable of forming balanced clusters, subject to restrictions, it allows us to build clusters of similar lengths, contributing in this way to reliable data on each cluster. With it, it is possible to make a sound planning with the respective analysis, which is part of a tree-type wireless hierarchical network. It is known that the above-mentioned conventional algorithm uses divisive methods to form clusters without observing the lengths of each one. Therefore, they are unpredictable and do not build balanced conglomerates. In addition, they are not able to accept design parameters such as: capacity and coverage.

**Figure 3:**
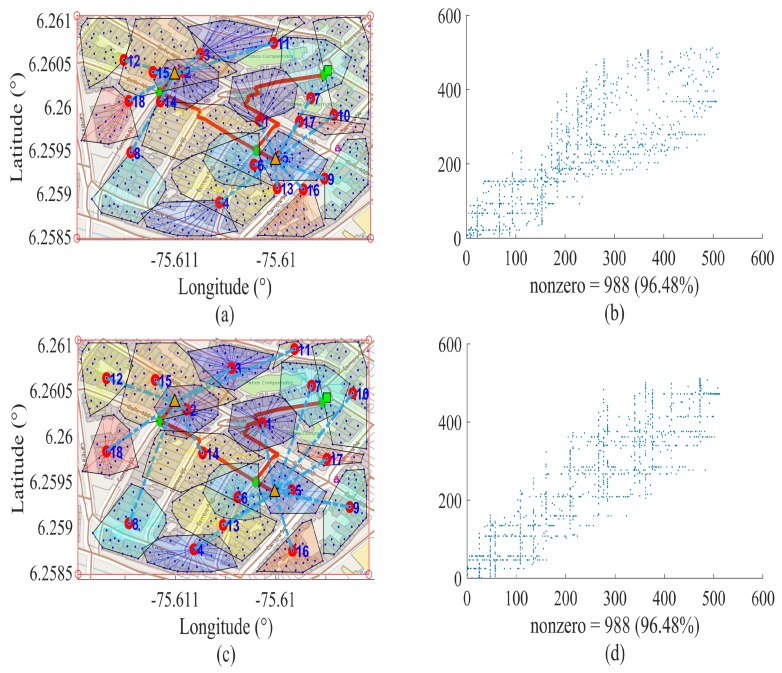
WiFi neighbor adjacency matrix *n* = 512. (**a**) and (**b**) preliminary deployment, (**a**) route map and (**b**) representation of the adjacency matrix; (**c**) and (**d**) correspond to the scenario, minimizing the delays. Source: the authors.

In Figure 3, we can identify the near optimal route map accompanying the respective sparsity pattern matrix (spy) obtained from the binary array of dispersed adjacency of length *n* × *n*. Therefore, using these square matrices, the binary relationships one and zero are represented, where one represents the existence of an edge and zero its non-existence. For each node, which binds to an edge, is placed a one represented in blue in Figure 3, and in the remainder is placed a zero represented in white color. Therefore, spy is a binary matrix that contains the information of the vertices and edges of the solution to the problem posed in this research. In this figure is proposed a scenario defined by a finite number of nodes, in which two different criteria of selection of the UDAP are applied. Figure 3a,b corresponds to the first criterion of the selection of the UDAP, that is by the minimum distance from the closest BS to one of the SMs of the corresponding cluster. The SMs that meets this condition will be selected as UDAP, and the rest will be single-access SMs to WiFi technology. Figure 3c,d corresponds to the second criterion, which applies the ODB algorithm for the selection of the UDAP. The characteristics of the sparsity pattern matrix in this research are: square matrix, binary, symmetric and the inputs of the zero diagonal. If the diagonal is zero, this is because there cannot be one edge of one vertex and toward the vertex *v*, since it will be the same vertex and it is not possible to construct a graph *G(V,V)*. Therefore, a graph is defined as *G(V,A)*, where *V* is the vertex represented by SMs, UDAPs, BSs or the central office, and A are the edges represented by the WiFi-cellular links that provide a link address, in such a way that a direct graph will be built. Therefore, the spy matrices in Figure 3 represent the connectivity array from a vertex *i* to a vertex *j*, denoted as *Vij*. The number of nonzero elements of the spy arrays is 988 (see Figure 3), which divided to two, results in 494, which is the number of WiFi links required by the network, which represents 96.48% of the use of technology with cost $C_{2}$ and 3.52% of cellular links at a cost $C_{1}$ for hybrid wireless communication. If we checked Scenario 1 in Table 3 and Table 4, we can identify that we need 494 WiFi links and 18 UDAPs, giving as a result *n* = 512, which is the number of SMs to deploy in *A(n)*. Accordingly, the number of nonzero elements of the spy arrays of Figure 3 corresponds to the set of vertices and edges $V_{ij}$ and its respective image $V_{ji}$, after which being added, we have $V_{ij}+V_{ji}$, if $V_{ij}=V_{ji}$; as we refer to the same link, the result is $2V_{ij}$. Therefore, if we replace the required number of WiFi links from Scenario 1 of Table 3 in the previous expression, we are left with the number of nonzero elements nonzeros=2∗494=988, presented in Figure 3.

Considering the above statements, in Figure 3b,d, completely different arrays can be seen, with the same number of nonzero elements, which correspond to the binary matrices resulting from adjacency by applying different criteria for the selection of the UDAP. In Figure 3b, greater dispersion of the nonzeros in the positions (400, 400) can be seen. Comparing this with Figure 3d, the existence of a greater number of jumps required to guarantee the coverage for each SMs available on the stage occurs; therefore, the dispersion is associated with the number of hops. Consequently, the end to end delay parameters and FSPL will be increased. In Figure 3d, through the application of the ODB algorithm, unnecessary dispersions are eliminated. Reducing the possible utilization of jumps to the maximum, to transmit data packages from the most distant SMs toward their respective UDAP, this contributes to a significant reduction of the delay for a UDAP to add and to merge the information of its associated clusters to relay to the respective BSs. In the same way, FSPL is diminished. In Figure 3, it can be determined that the SMs suitable to be selected as UDAP by the ODB algorithm are the nearest nodes to the center of mass of each group. Thereby reducing the average end to end delay of each group to the maximum. This decreases the average number of links that a data package must pass through to reach the respective UDAP. If the number of crossed links increases, this is because the SMs are far away from their respective UDAPs and require mandatory jumps to be able to transmit. This can happen because the radio coverage of the UDAP does not guarantee observability of the furthest SM. Therefore, if the number of crossed links to transmit data packages from SMs until their respective UDAP increases, this is because in the same way, different variables increase, such as the distances of transmission and the jumps required, and consequently, end to end delay increases. Therefore, the end to end delay is directly proportional to the number of average links crossed by a data package.

In addition, through Figure 3, it is shown that the heuristics proposed is able to mutate the adjacency matrix, seeking to provide the best resulting topology to the solution of the problem. The topology will ensure a significant reduction of the average end to end delay that the UDAP takes to add the information of its associated clusters. Therefore, in Figure 3c,d, the georeferenced near optimal deployment of SMs is shown. This serves for measurement, monitoring and control of the conventional electrical system, giving rise to the possibility of an optimal data management and the integration of micro-grids to increase the reliability and quality of energy.

Table 3 presents the required number of links and the computation of the analyzed variables in this paper for the required wireless WiFi network. It presents five different scenarios, in which the density of SMs is varied *n* (512, 256, 128, 64 and 32) to be deployed in A(n), thus demonstrating the criterion of scalability enabled by the heuristic proposed. It is known that *n* is the sum of WiFi and cellular links and can be confirmed in the corresponding scenarios using Table 3 and Table 4. The purpose of these tables is to quantify the necessary resources and review the behavior of the network in its different scenarios by analyzing the number of WiFi links and cellular links required, coverage rates, average maximum distances of intra-cluster and inter-cluster links, average time that a UDAP takes to add the information and the computation of FSPL considering different frequencies applicable to a wireless WiFi and cellular network. Each of these results allows us to plan the deployment of the network by observing their behavior. Considering that by the proposed heuristic, the minimum values on FSPL, end to end delay and transmission distances are obtained, this provides a near-optimal solution to the planning problem exposed in this research.

As the frequency of the wireless WiFi and cellular network signal increases, also the FSPL metrics increase. In general terms, the lower the frequency of transmission, the better will be the signal that will travel through the air and the objects. FSPL is used to predict the intensity of the required signals in a wireless system. In addition, in Table 3 and Table 4, if we add the delays that it takes a UDAP to collect the information of the cluster and the delay in a cellular technology, we can estimate the average total time in which the BSs have the data of each UDAP deployed in the scenario merged available. The data of Round Trip Time (RTT) of Table 4 are taken from [82], which are applied in cellular technology. If we compare Table 3 and Table 4, we can see that the metrics of delays in WiFi are much greater than the metrics of cellular delays. However, the amount does not exceed the allowed delays in AMI exposed in the literature for efficient data aggregation.

Therefore, with Table 3 and Table 4, viewing each scenario, we can obtain the required procedures for the deployment of SMs under the configuration of a hybrid wireless network (WiFi-cellular). Another fact of much interest is the length of optical fiber between the BSs and the central office. In this case study, the length is 280 m in all scenarios, since the latitude and longitude coordinates of the BSs and central office are fixed. As a result, the heuristic has been able to provide a minimum route map, required for the planning of a hybrid FiWi network at the lowest cost while maximizing reliability and the robustness of the bidirectional communication network needed to control and supervise the conventional electrical network allowing us by optimal information management to integrate SMG systems that will be able to run connected to the network through an adequate synchronization and, in the same way, able to work in islanded mode, namely disconnected from the system. The importance of microgrids, through an adequate two-way communication system, is that they can operate autonomously according to what the physical and economic conditions dictate.

**Figure 4:**
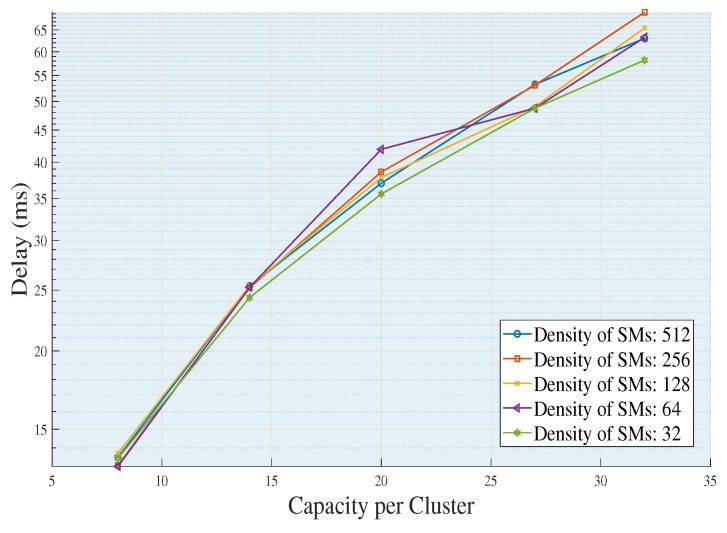
End to end delay generated by each population increase by varying the capacity of each cluster with traffic 0.1 package/s, *L* = 200 bits. Source: the authors.

Figure 4 shows the increases in end to end delays as the capacity of a UDAP to accommodate SMs increases. This happens because the ability to agglutinate a cluster is directly related to the number of average links that a data package must go through to transmit the package from the SMs to their respective UDAPs. In addition, the higher the capacity of the UDAP, the more various effects may occur, such as: increased delay time in collecting the information, greater distances of transmission, greater number of jumps and greater chargeability of each link in the network. On the other hand, in each density of SMs, the topologies of each cluster are changing, to comply with the requirements of the network, which causes and requires different routing characteristics to the extent that the density of SMs is increasing or decreasing, causing variability in the features of each cluster and therefore the resulting topology. As a result, if clusters are built with minimum distances, the need to transmit through multiple jumps is null. Therefore, the delay is directly proportional to the capacity-coverage of the UDAP and inversely proportional to the density of the SMs.

In Table 5, the capacity algorithm is presented 2. This algorithm helps to reduce the average times in which the UDAP collects the information from the group. The ODB algorithm performs intra-cluster scans to determine the best concentrator position (UDAP). It can be seen that by increasing the density of nodes (SMs) and maintaining the capacity of the conglomerates, the need to deploy UDAPs also increases. The number of UDAPs required in each scenario is different since a heuristic has a stop criterion. Therefore, once the restrictions are met, the algorithm stops providing one of the possible combinations as a solution that satisfies the constraints of the problem. Moreover, the model being combinatorial and having complexity that is NP-complete only provides solutions that are close to optimal. Hence, it would demand an excessive computational time to explore each of the possible combinations and, thus, to determine an optimal global solution. Consequently, the stopping criteria (restrictions) contribute to the relaxation of machine time that the heuristic takes to provide a near optimal solution. Finally, Table 5 shows the end to end transmission percentages that can be reduced by applying the ODB algorithm to a previous solution.

**Figure 5:**
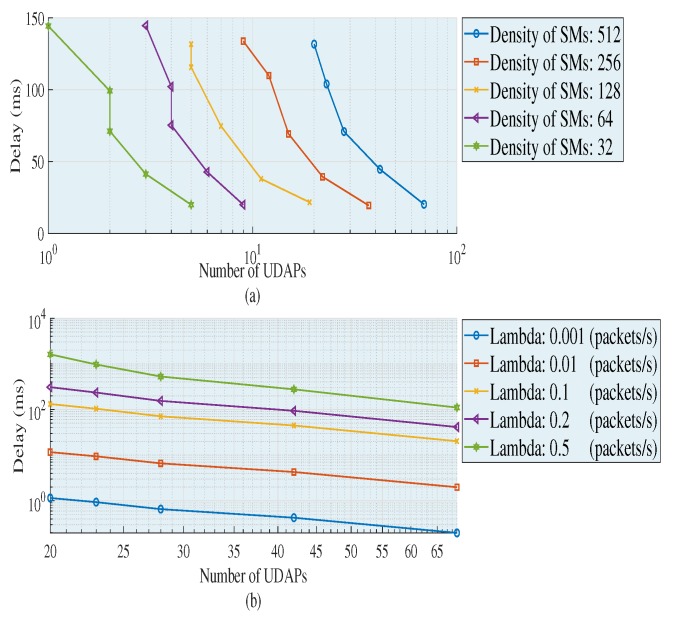
Delay in different scenarios. (**a**) Delay vs increase of users; (**b**) Delay vs increase packet rate. Source: the authors.

Figure 5a shows the metric obtained with the following characteristics: data length *L* = 800 bits, Lambda = 0.1 package/s and by varying the density of SMs and the capabilities of each cluster. In Figure 5b, *L* is kept, the density of SMs is *n* = 512 and Lambda and the capabilities are varied. In Figure 5a,b, it is noted that, when the need of UDAPs decreases, the average delay of the entire wireless network increases. This happens due to the increase in the capacity of each UDAP to accommodate SMs. If the capacity to accommodate SMs of a UDAP increases and its radio coverage is minimum, the need for multiple jumps to aggregate data from the more distant nodes to the UDAP also increases. Therefore, as the multiple jumps in the cluster increase, there is also an increase in the distance of an SM to its associated UDAP. This translates into an increased time required to add and merge the data in each UDAP. In addition, in Figure 5a, it can be seen that the average delays while maintaining the capacity are similar in each increment of density of the deployed SMs. This is because these are partial averages of each cluster, which demonstrates that the heuristics is capable of building, through appropriate topologies, balanced graphs, which in turn directly contributes to decreasing technical losses in a wireless network. Therefore, the amount of required UDAPs responds to three variables in particular: Density of SMs, capacity and coverage (in terms of the technical characteristics available for the UDAP).

If we verify the behavior of the metrics in Figure 5a, in the populations of 32 and 128 with capacities of 20–27 and 27–32, respectively, there is no need to implement a UDAP since the proposed algorithm searches in each capacity increment to include (if the capacity allows it) the nodes that were not included (due to the restrictions of the problem); thereby completing the clusters without the need of adding UDAPs. On the other hand, in Figure 5a, it is clear that as the SMs’ density increases, the slope of the delays is stabilized. This happens because, as it has a larger number of SMs, the algorithm manages to build clusters mostly balanced in terms of the following: distances, radio coverage and number of elements for each group. Therefore, the higher the density of SMs, the better the results obtained in terms of optimization due to the closeness of the SMs. Therefore, when varying the capacities of a UDAP, the following is modified: the topology, the average number of traversed links by the package to reach its destination, the length of the cluster, the end to end delay, the link capacity and the coverage distance.

In Figure 5b, significant variations in the global delay are depicted for the data aggregation as the traffic generated by each SM increases. Therefore, the higher the traffic generated, the greater the FiWi network delay. This is because the increase in delay is directly proportional to the increase in capacity. If the capacity of the UDAP increases, the greater will be the length of the cluster, and therefore, the greater will be the traffic generated in each cluster; resulting in an increase in the global end to end delays. Accordingly, the delay is directly proportional to the traffic generated by each SMs, whereas the number of UDAPs *k* required is inversely proportional to the capacity and coverage of the UDAP.

**Figure 6:**
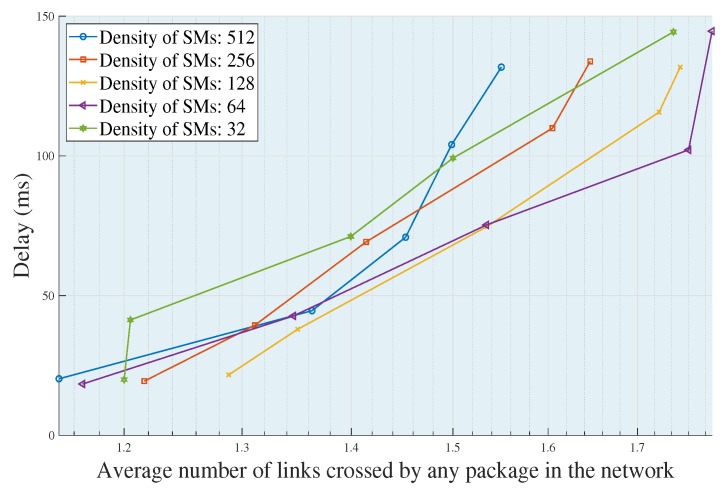
Average links crossed by a data packet *L* = 800-bit, Lambda = 0.1 package/s. Source: the authors.

In Figure 6, it is shown that the greater the amount of average links that a data package must go through from an SM source to a UDAP, the greater the increase in the delays of each scenario. This happens due to the following reason: if the average number of links that a data package must go through increases, this is because the package was generated by a node that is located at a greater distance than the maximum radio coverage allowed for the UDAP. That is, if a node is very distant, it increases the global delays of the wireless network. Due to this, the data package has to carry the information through jumps, supported on the SMs of transition, to bring the information to the UDAP. Each trend in Figure 6 corresponds to a different scenario. Therefore, the behavior of each trend responds to the near optimal topology in each of the cases. This heuristic is a solution to the problem of planning.

If we see the trend with *n* = 512, in Figure 6, we can corroborate the affirmation made in previous paragraphs: the higher the number of deployed SMs, the better the optimization results reached. Therefore, in Figure 6, it is shown that when there is a high density of SMs, the average number of jumps required for the transmission of data packages is lower than in all other cases. This is because the greater the number of SMs, the more dispersions are avoided (see Figure 3). Consequently, this translates into technical losses in a wireless network. Finally, if the average of links crossed by a package is zero, this means that the entire network does not require multiple jumps to transmit the information from a source SM to a target UDAP.

## 5. Conclusions

The heuristic proposed allows practitioners to deploy the necessary number of UDAPs for the monitoring, supervision and control of conventional electrical network, providing coverage to a number *n* of SMs and making possible the integration of microgrids with the conventional electrical system. In this way, final users of energy resources will become consumers and prosumers thanks to the integration of DRES. A fundamental feature of the model is that it adapts to the conditions of the required wireless network. In addition, the research carried out allowed us to determine the importance of reducing to the maximum the end to end delay of the entire network. This metric not only provides information in terms of time, but in addition, allows us to comprehend and minimize the chargeability of the network and the need to allocate the capacity of the point-to-point links for its efficient operation. The model has been shown to be scalable in time and space and has the following characteristics: presents finite solutions and optimizes the resources required by the FiWi network using an efficient clustering method (different to the traditional). Moreover, with the N-NST algorithm, balanced clusters can be built, which are subject to real restrictions, such as capacity and coverage. The heuristics works with georeferenced scenarios, reducing to the maximum the aggregation delays of data of each cluster using the ODB algorithm. Furthermore, it minimizes FSPL and is a planning model of NP-complete complexity. The complexity of the problem lies in the population density of SMs, since, in a graph with *n* SMs, there are $n^{n-2}$ possible trees; thus, the proposed model is combinatorial in nature. Hence, the results obtained are near optimal due to the exponential increase in the complexity if there is a minimal increase of the SMs in the scenario.

Consequently, in order to relax the problem, stop criteria are introduced. The goal is that once the algorithm converges, it stops providing a near optimal solution. We assume that all the nodes are linked by cellular technology, a very expensive situation. As the model replaces the cellular links with lower cost WiFi links, the objective function decreases as much as possible, thus approaching the optimal solution. Once the model cannot further decrease the cost, the algorithm stops. Therefore, the objective of this research is to minimize cellular links and to maximize WiFi links guaranteeing coverage to the nodes located in the area of interest. Another fundamental characteristic of the present model is its combinatorial nature; because, if the density of nodes increases and due to the capacity and coverage restrictions, the nodes are not covered, and after verifying the best options, these nodes must necessarily be UDAPs and could serve as future expansions.

In future works, a comparative analysis will be carried out between different clustering methods. The link capacity restriction (Mbps) will be increased to decide on the topology, and finally, the fault tolerance will be included, as well.

## Figures and Tables

**Table 1:** Simulation model and parameter.

Item	Parameter	Value
Deployment	Node density	4734 nodes/km${}^{2}$
	Node placement	Georeferenced
	No. of nodes per cluster	m {8, 14, 20, 27, 32}
	Coverage WiFi	$r_{ds}$ 60 m
	Coverage cellular	$r_{db}$ 1000 m
PHY	Standard	IEEE 802.11b
	Frequency band	2.4 GHz
	Transmission rates	{0.5, 1, 2, 5, 11} Mbps
MAC	Standard	IEEE 802.11 b
		3 G, 4 G, 5 G
	Operation mode	Tree
APP	Application layer data length	L 100 bytes/packet
	Packet rate	Lambda {0.001, 0.01, 0.1, 0.2, 0.5} packets/s

**Table 2:** Variables used. SM, Smart Meter; FSPL, Free Space Path Loss; ODB, Optimal Delay Balancing.

Nomenclature	Description
$SM_{\;x,y}$	Coordinates, longitude and latitude for SMs
$BS_{\;x,y}$	Coordinates, longitude and latitude for the base station
$C_{off\;x,y}$	Coordinates, longitude and latitude for the central office
$\gamma_{i,j}$	Vector of pairs of adjacent nodes
β	Vector preliminary computation of end to end delay and FSPL
α	Result vector of end to end delay and FSPL calculated with the ODB algorithm
*n*	Number of smart meters
*A*	Georeferenced area
*Z*	Universal data aggregation point
*X*	Smart meters
*G*	Adjacency matrix
*m*	Capacity restriction
*s*	Length cluster
*k*	Number of clusters
*w*	Maximum number of hops allowed
*h*	Hop number counter
$C_{1},C_{2},C_{3}$	Unit costs, cellular, WiFi and optical fiber
$C_{wf},C_{cell},C_{fop}$	Total costs, WiFi, cellular and optical fiber
$r_{ds},r_{db}$	WiFi and cellular coverage restriction, respectively
$r_{ni},r_{ns}$	Haversine distance (m) of the intra- and inter-cluster
$dist_{i,j}$	Haversine distance matrix *n* × *n*
$d_{fop}$	Distance (m) of optical fiber

**Table 3:** Wireless WiFi network: *L* = 800-bit/packet, Lambda = 0.1 package/s. Source: the authors.

Scenario	WiFi	Coverage	Distance (m)	Delay Cluster (ms)	Parameters FSPL (dB)		
#	# of Links	%	Average	Average	2.4 GHz	5.4 GHz	5.8 GHz
1	494	100	30.12	228.26	69.63	76.68	77.30
2	245	100	30.27	192.85	69.67	76.82	77.84
3	124	100	33.44	267.65	70.54	77.60	78.20
4	62	100	31.98	258.29	70.15	77.20	77.82
5	31	100	25.52	236.95	68.19	75.24	75.86

**Table 4:** Wireless cellular network. Source: the authors.

Scenario	Cellular	Coverage	Distance (m)	Rand Trip Time (ms)			Parameters FSPL (dB)		
#	# Links	%	Average	3 G	4 G	5 G	850 MHz	1700 MHz	1900 MHz
1	18	100	84.23	70	20	5	69.55	75.57	76.53
2	11	100	59.46	70	20	5	66.52	72.54	73.51
3	4	100	55.03	70	20	5	65.85	71.87	72.84
4	2	100	68.41	70	20	5	67.74	73.76	74.73
5	1	100	66.76	70	20	5	67.53	73.55	74.52

**Table 5:** *L* = 200 bit/packet, Lambda = 0.1 packet/s, capacity = 32. Source: the authors.

Scenario	Density of SMs	UDAPs	Delay without ODB Algorithm	Delay with ODB Algorithm	
#	*n*	Units	Average End to End (ms)	Average End to End (ms)	Reduce %
1	512	18	67.17	55.60	17.25
2	256	11	53.96	46.73	14
3	128	4	75.37	65.09	13.65
4	64	2	82.61	62.88	23.88
5	32	1	72.98	57.89	20.68
